# Accumulation of Heavy Metals in Tea Leaves and Potential Health Risk Assessment: A Case Study from Puan County, Guizhou Province, China

**DOI:** 10.3390/ijerph15010133

**Published:** 2018-01-13

**Authors:** Jian Zhang, Ruidong Yang, Rong Chen, Yishu Peng, Xuefeng Wen, Lei Gao

**Affiliations:** 1 College of Resource and Environmental Engineering, Guizhou University, Guiyang 550025, China; jzhanggzdxhjkx@163.com (J.Z.); pengys520@126.com (Y.P.); gaoleixueshu@163.com (L.G.); 2 College of Mining, Guizhou University, Guiyang 550025, China; rchengzu@163.com; 3 College of Agriculture, Guizhou University, Guiyang 550025, China; wenxuefeng@aliyun.com

**Keywords:** heavy metal, soil, tea leaves, bioconcentration factor, potential health risk

## Abstract

This study features a survey of the concentrations of aluminum (Al) and heavy metals (Mn, Pb, Cd, Hg, As, Cr, Ni, Cu, and Zn) in tea leaves and the corresponding cultivation soils (0–30 cm), carried out in Puan County (Guizhou Province, China). The average concentrations of Al, Mn, Pb, Cd, Hg, As, Cr, Ni, Cu, and Zn in the soil were 106 × 10^3^, 214, 20.9, 0.09, 0.12, 17.5, 121, 27.8, 131.2, and 64 mg·kg^−1^, respectively. The heavy metals’ pollution indexes in the soil can be ranked as follows: Cu > Cr > Hg > As > Ni > Zn > Pb > Mn > Cd. The soil was moderately polluted by Cu because of the high geochemical background value of Cu in the area. The potential environment risk index (RI) showed that 7.69% out of the total sample sites were within the moderate level. Moreover, the ranges of Al, Mn, Pb, Cd, Hg, As, Cr, Ni, Cu, and Zn concentrations in young tea leaves were 250–660, 194–1130, 0.107–0.400, 0.012–0.092, 0.014–0.085, 0.073–0.456, 0.33–1.26, 6.33–14.90, 14.90–26.10, and 35.8–50.3 mg·kg^−1^, respectively. While in mature tea leaves, they were 4300–10,400, 536–4610, 0.560–1.265, 0.040–0.087, 0.043–0.089, 0.189–0.453, 0.69–2.91, 3.43–14.20, 6.17–16.25, and 9.1–20.0 mg·kg^−1^, respectively. Furthermore, the concentrations of Pb, Cu, As, Hg, Cd, and Cr in young tea leaves and mature tea leaves were all lower than the standard limit values (5.0, 30, 2.0, 0.3, 1.0, and 5.0 mg·kg^−1^ for Pb, Cu, As, Hg, Cd, and Cr, respectively) in China. Besides, the accumulation ability of tea leaves to Mn was the strongest, and the average bioconcentration factor (BCF) of Mn in mature tea leaves was 12.5. In addition, the average target hazard quotients (THQ) were all less than one for the young tea leaves and the average aggregate risk hazard index (HI) to adults was 0.272, indicating that there was not a potential health risk for adults through the consumption of the infusions brewed by young tea leaves. However, for mature tea leaves, the percentage which HI values were above one was 38.46%, and the risk to adults via the consumption of mature tea infusions were mainly contributed by Mn and Al.

## 1. Introduction

Tea is one of the most popular alcohol-free and caffeinated beverages in the world. It is made from new tea leaves and then brewed with boiling drinking water to get a tea infusion. Tea trees are mainly grown in some Asian and African countries, such as China, India, Sri Lanka, Kenya and Zimbabwe, etc. [1]. Because tea contains tea polyphenols (catechins), amino acids, tannic acid, and other antioxidants drinking tea is considered beneficial to human health, including the prevention of many diseases since it has been proven to prevent Alzheimer’s disease, high blood pressure, and obesity [2]. In addition, the essential trace elements in humans can be supplemented through drinking tea because tea leaves contain potassium, manganese, selenium, boron, zinc, strontium, and copper [3].

The quality of tea is important for the development of the tea industry, tea farmers’ income, and the health of tea drinkers. In particular, heavy metals in tea are important indicators in the process of tea quality evaluation, as they can be transferred into tea infusions through the process of brewing tea, then enter the human body by means of tea consumption, and thus pose potential risks to human health. Thus, the chemical components in tea, particularly heavy metals, have received great interest because they are related to human health. According to the national food safety standard (GB 2762-2017) [4] and the Ministry of Agriculture tea heavy metals limited standards (NY/T 288-2012, NY 659-2003) in China [5,6], the standard limit values are 5.0, 30, 2.0, 0.3, 1.0, and 5.0 mg·kg^−1^ for Pb, Cu, As, Hg, Cd, and Cr, respectively. It was reported that heavy metals (Pb, Cd, Cr, As, Mn, Hg, and Cu) pollution has occurred in some tea garden soils in recent years as a result of high soil background values, the application of pesticides and chemical fertilizers containing heavy metals, and industrial activities [1,7,8]. The mechanism of heavy metal enrichment in tea trees is very complex, and is related to the age and organs of tea trees, soil physico-chemical properties, the elements’ properties, contents, and the speciation [9,10]. For instance, heavy metals found in Puerh tea from Yunnan Province, China, did not pose a carcinogenic risk to humans, but the carcinogenic risk of As should be of concern [11]. Moreover, the average bioconcentration factor (BCF) of Mn in mature tea leaves was 5.3, indicating the accumulation capacity of Mn in tea leaves is very strong, and the health risk of metal elements in tea from South Anhui Province mainly came from the chemical carcinogen Cr [12]. Similarly, the concentrations of Mn in tea leaves and infusions of different types tea were the highest among five heavy metals, and the studied heavy metals could be arranged in descending order according to their contents in tea brew as follows: Mn > Fe > Zn > Cu > Pb [13]. The concentrations of major heavy metals (As, Cr, Cd, and Pb) were significantly different (*p* < 0.05) among the ten famous teas from China’s Zhejiang Province, but lower than the relevant tea standards limit values in China, indicating that the quality of tea in Zhejiang Province was good [14]. In addition, Shi et al. [15] reported that the transfer abilities of As and Cd from the root to the upper part in tea trees were slow, and the tolerance of tea plants to As was stronger than that of Cd when the soil in a tea garden was polluted. Besides, the results of some studies suggest that tea drinkers should discard the first infusion and instead consume the following infusions, because the combined health risk associated with heavy metals in the first infusion was higher than those of the second and later infusions [1,16]. In the latter case, the target hazard quotient (THQ) values of each metal through the consumption of tea were all below one, but the hazard index (HI) values, with 2 out of 15 tea samples, were found to be greater than one and this was an indication of a significant non-carcinogenic health risk to consumers [17].

Tea is thought to have originally derived from Guizhou Province (China), and there is a long history of tea planting in Guizhou Province. Specifically, the “tea seed fossil” more than one million years old was discovered in Yuntou Mountain in Puan County (Guizhou Province, China). By the end of 2016, the tea planting area in Guizhou Province was over 4.67 × 10^5^ hectares, and the planting area has been ranking the first in China for several years. Furthermore, the ecological environment in Guizhou Province was favorable to tea production and characterized by low latitude, high attitude, little sunshine, and many clouds. Many literature studies have focused on the concentrations of heavy metals in tea and soil in Guizhou. As an example, Zhang et al. [18] found that the contents of heavy metals (Cd, Hg, As, Pb, Cr, and Cu) in soil were higher in three regions (Duyun, Meitan, and Guiding) in Guizhou, but the heavy metal contents in tea were below the safety standards in China. Moreover, the bioconcentration factors (BCF) of Hg and Cu in tea leaves were higher, and there were higher leaching rates in the tea-tea infusion system, which caused Hg and Cu to be transferred easily in the soil-tea-tea infusion system in the Yunwu area (Guiding County, Guizhou Province) [19]. However, there are far fewer studies on the contents of heavy metals in soil and tea leaves and health risk assessment in the Puan tea area of Guizhou Province. Merely, the contents of selenium (Se) in soil and spring tea leaves were reported in this region [20]. On the other hand, tea plants have been demonstrated to be Al accumulators due to the acidic soils where tea plants are grown [1]. Therefore, the identification of heavy metals and Al contents in soil and tea leaves in the Puan tea area of Guizhou Province and a human health risk evaluation of drinking tea were deemed necessary as this data is of significance for the sustainable and healthy development of tea industry in Puan County, Guizhou Province, China.

Consequently, the objectives of the present study were to: (1) measure the concentrations of Al, Mn, Pb, Cd, Hg, As, Cr, Ni, Cu, and Zn in tea leaves (young and mature) and the corresponding soil by using inductively coupled plasma mass spectrometry (ICP-MS) and inductively coupled plasma atomic emission spectrometry (ICP-AES); (2) discuss the bioconcentration factors (BCFs) of heavy metals and Al in young tea leaves and mature tea leaves and analyze influencing factors; and (3) assess the potential health risks of these ten elements in young tea leaves and mature tea leaves to human using the target hazard quotient (THQ) and hazard index (HI) methods.

## 2. Materials and Methods 

### 2.1. Study Area

**Figure 1:**
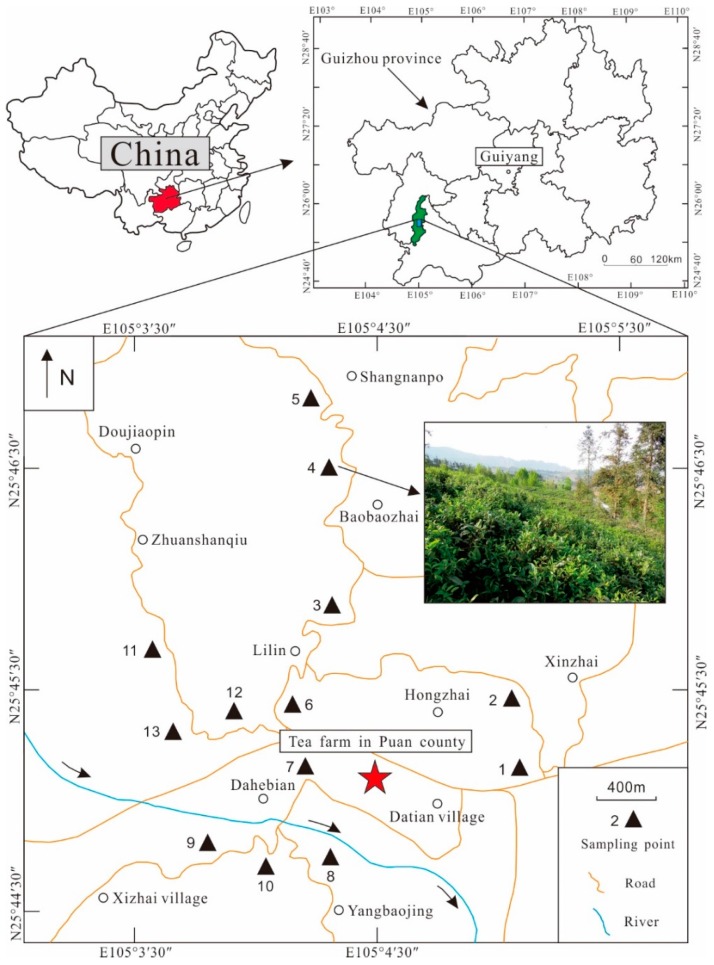
Study area and location of the sampling points for soil and corresponding tea leaves collection.

The study area (Puan County) which lies at 25°28′31″–26°10′35″ N and 104°51′10″–105°09′24″ E is located in southwest Guizhou Province (Figure 1), southwestern China. The altitude ranges from 1000 m to 1300 m. The average annual temperature is about 14 °C, with an average annual sunshine duration of 1563 h and an average annual precipitation of 1395.3 mm [20]. Puan is considered the birthplace of tea in the world, and is known as “the hometown of Chinese ancient tea trees” and “the hometown of Chinese tea culture”. Tea trees are mainly cultivated on the upper Permian Longtan Formation, and the bedrock is composed of terrestrial siliceous clastic rocks, such as sandstone, mudstone, and shale. Furthermore, the soil type is sandy yellow soil. The different tea varieties grown in Puan tea growing area include China Yunnan big leaves tea, Kunming little leaves tea, Fuding big white tea, Wuniu zao tea, and Longjing 43.

### 2.2. Sample Collection and Pretreatment

A total of 13 soil samples (0–30 cm) were collected in April 2016. Approximately 1.0 kg of soil was taken for each site and grass, leaves, roots and stones were manually removed. Meanwhile, 13 corresponding young tea leaves samples and 13 mature tea leaves samples were collected. Both the young and mature tea leaves samples were approximately 0.5 kg in weight. The information of sampling points was recorded using the global positioning system (GPS). A sampling point diagram is shown in Figure 1.

All soil samples were dried in a thermostatic air-blower-driven drying closet at 40 °C to a constant weight, redundant roots and stones were removed, and the samples were ground, passed through a 200-mesh nylon sieve (0.074 mm) and thoroughly homogenized in the lab, waiting for analysis. All fresh tea leaves samples were directly washed three times with tap water to remove dust and particles adhered to the tea leaves samples, and then washed three times with ultrapure water (18.2 MΩ·cm, 25 °C). Then, these samples were dried in a thermostatic air-blower-driven drying closet at 45 °C until a constant weight was reached. Subsequently, these tea samples were ground using a portable high speed universal grinder (100 g, DFT-100, Linda Machinery Ltd., Wenling, Zhejiang Province, China), sieved with a 200-mesh nylon sieve (0.074 mm), and then placed in polyethylene plastic bags for further chemical analysis.

### 2.3. Chemical Analysis

Soil pH was measured in ultrapure water (soil:water 1:2.5 *w*/*v*) with a PHS-3E pH meter (Shanghai INESA Scientific Instrument Incorporated Company, Shanghai, China). The analysis of tea leaves samples and soil samples was performed in an accredited laboratory (ALS Minerals-ALS Chemex Co., Ltd., Guangzhou, China) using ICP-MS (7700x, Agilent, Santa Clara, CA, USA) and ICP-AES (Vista-MPX, Agilent). The operation parameters of ICP-MS and ICP-AES in this study were set as proposed by Li et al. [1]. In the process of tea samples analysis, a 1.0 g tea sample was accurately weighted and added with 5 mL concentrated HNO_3_ into a Teflon digestion vessel and digested slowly at room temperature for about 8 h, before heating for 3 h in the graphite furnace. After cooling, the sample was dissolved and transferred to the volumetric flask, and the digestion solutions were diluted to a constant volume (25 mL) with 2% hydrochloric acid (HCl). Moreover, 0.25 g soil samples were digested by HClO_4_-HNO_3_-HF-HCl and diluted to a constant volume (12.5 mL) with 2% hydrochloric acid (HCl). On the other hand, 0.5 g soil samples were dissolved by aqua regia and diluted to a constant volume (12.5 mL) with deionized water. All acids used were ultra-pure grade. For quality assurance and quality control (QA/QC), the method blanks, duplicates and standard reference materials (GL03 and SpL02 representing plant samples; GBM908-10, MRGeo08, OGGeo08, GLG908-4, OREAS-45d, and OREAS-45e representing soil samples) were analyzed.

For the tea samples determination method, the limits of detection (LOD) values of Al, Mn, Pb, Cd, Hg, As, Cr, Ni, Cu, and Zn were 10, 0.1, 0.005, 0.001, 0.001, 0.005, 0.05, 0.02, 0.01, 0.1 μg/g, respectively. The upper limits values of detection of Al, Mn, Pb, Cd, Hg, As, Cr, Ni, Cu, and Zn were 2.5 × 10^5^, 5.0 × 10^4^, 9.0 × 10^3^, 2.0 × 10^3^, 1.0 × 10^2^, 1.0 × 10^4^, 1.0 × 10^4^, 9.0 × 10^3^, 9.0 × 10^3^, 9.0 × 10^3^ μg/g, respectively. Furthermore, the mean recoveries for these 10 elements (C (element, measured)/C (element, certified) × 100%) in the two standard reference materials (SRMs) were between 90% and 113%. Moreover, the duplicate samples showed that the relative deviation (RD) ranged from 0.00% to 8.15% (<10%), indicating the tea samples are thoroughly homogenized. The test values of blank samples were all lower than the detection limits. Hence, the quality of the analysis data was reliable.

For the soil samples analytical method, the limits of detection (LOD) values of Al, Mn, Pb, Cd, Hg, As, Cr, Ni, Cu, and Zn were 100, 5, 0.5, 0.02, 0.004, 0.2, 1, 0.2, 0.2, 2 μg/g, respectively. The upper limits values of detection of Al, Mn, Pb, Cd, Hg, As, Cr, Ni, Cu, and Zn were 5.0 × 10^5^, 1.0 × 10^5^, 9.0 × 10^3^, 1.0 × 10^3^, 1.0 × 10^2^, 1.0 × 10^4^, 1.0 × 10^4^, 5.0 × 10^3^, 9.0 × 10^3^, 1.0 × 10^4^ μg/g, respectively. Besides, the mean recoveries for 10 elements (C (element, measured)/C (element, certified) × 100%) in the six standard reference materials (SRMs) were between 91% and 108%. The duplicate samples showed that the relative deviation (RD) ranged from 0.00% to 9.76% (<10%), indicating the soil samples are thoroughly homogenized. The test values of blank samples were all lower than the detection limits. Therefore, the quality of these soil concentrations data was guaranteed.

### 2.4. Soil Pollution Quantification Assessment

#### 2.4.1. Geo-Accumulation Index

The geo-accumulation index (*I_geo_*) has been widely used to assess the soil contamination status with heavy metals [21,22,23]. This method is a kind of quantitative index to study the degree of heavy metal pollution in soil, sediment, and urban street dust. In present study, this effective method of *I_geo_* was applied to assess the pollution degree of heavy metals in Puan tea area [24]. The *I_geo_* was calculated by using the following Equation (1):

*I_geo_* = log_2_[C_n_/(1.5 × B_n_)] (1)

where *I_geo_* is the index of geo-accumulation of n element; C_n_ is the metal content (mg·kg^−1^) in the investigated surface soil samples; B_n_ is the surface soil layer (0–20 cm) background value (mg·kg^−1^) of this element in Guizhou Province [23,25]; and coefficient of 1.5 is the factor to minimize the effect of possible lithogenic variations in soil [21]. According to Müller [24], the following criteria based on the values of *I_geo_* were applied to assess the metal contamination level in the soil:

*I_geo_* ≤ 0 Unpolluted;
0 < *I_geo_* ≤ 1 Unpolluted to moderately polluted;
1 < *I_geo_* ≤ 2 Moderately polluted;
2 < *I_geo_* ≤ 3 Moderately to heavily polluted;
3 < *I_geo_* ≤ 4 Heavily polluted;
4 < *I_geo_* ≤ 5 Heavily to extremely polluted;
*I_geo_* > 5 Extremely polluted.

#### 2.4.2. Enrichment Factor

The enrichment factor (EF) provides information about the normalized metal concentration in soil above the uncontaminated level. For normalization, a reference metal concentration is used. The most widely used reference element for this purpose is Al because of its natural abundance and less interaction with other heavy metals. The EF was calculated by using the following Equation (2) [26]:

EF = (C_n_/C_ref_)_sample_/(C_n_/C_ref_)_baseline_ (2)

where C_n_ and C_ref_ are the respective metal and reference element (Al) concentrations. Sample and baseline represent soil samples and background, respectively. In this study, the background values of A layer soil (0–20 cm) in Guizhou Province were used as background values [25,27]. By means of the correlation analysis, the correlations between Al and other heavy metals were the smallest, and the Al contents were the most stable in the soil samples. Hence, Al was chosen as the reference element. According to Sutherland [26], the following criteria should be employed to determine the metal enrichment in soil based on the EF values:

EF < 2 deficiently to minimal enrichment in soil;
2 ≤ EF < 5 moderate enrichment in soil;
5 ≤ EF < 20 significant enrichment in soil;
20 ≤ EF < 40 very high enrichment in soil;
EF > 40 extremely high enrichment in soil.

#### 2.4.3. Potential Ecological Risk Assessment

The potential ecological risk index is often used to assess the quality of the soil environment. The potential ecological risk index was calculated by means of the following Equation (3) [23,28]:

(3) $$\mathrm{RI}=\sum E_{r}^{i}=\sum T_{r}^{i}\times C_{s}^{i}/C_{n}^{i}$$

where risk index (RI) is the sum of potential ecological risk indices for all detected heavy metals; $E_{r}^{i}$ is the potential ecological risk index of an individual element; $C_{s}^{i}$ is the concentration of each element; $C_{n}^{i}$ is the background value (mg·kg^−1^) of the element in A layer soil from Guizhou Province [25]; and $T_{r}^{i}$ is the toxicity response factor for each element, which expresses the toxic levels and the environmental response (Zn and Mn = 1; Pb, Cu and Ni = 5; Cr = 2; As = 10; Cd = 30; Hg = 40) [23,28]. The boundary criterion was readjusted using the average RI value method of pollutants, which overcame the arbitrariness of the classification standard setting, and can reflect the practical situation. In present study the single ecological index is classified into five categories [28,29]:

$E_{r}^{i}$ < 40 low risk;
40 ≤ $E_{r}^{i}$ < 80 moderate risk;
80 ≤ $E_{r}^{i}$ < 160 considerable risk;
160 ≤ $E_{r}^{i}$ < 320 high risk;
$E_{r}^{i}$ ≥ 320 very high risk.

The integrated potential ecological risk index (RI) is classified into four categories [28,29]:

RI < 110 low risk;
110 ≤ RI < 220 moderate risk;
220 ≤ RI < 440 considerable risk;
RI ≥ 440 very high risk.

### 2.5. Bioconcentration Factor (BCF)

The bio-concentration factor (BCF) is an important quantitative indicator of crop contamination and has commonly been used for estimating metal transfer from soil to plants, since the concentrations of toxic elements were different with the change of the plant species. In particular, the bioconcentration factor (BCF) is a ratio of heavy metal concentration of plant to soil. It was carried out to evaluate the accumulation behaviors of toxic elements in tea leaves and calculated through the following Equation (4):

BCF = C_plant_/C_soil_ (4)

where C_plant_ and C_soil_ were the toxic element concentrations in tea leaves and the corresponding soil (mg·kg^−1^), respectively, on the basis of dry weight [22,30].

### 2.6. Health Risk Assessment

#### 2.6.1. Estimated Daily Intakes

The estimated daily intake (EDI) of Al and heavy metals is a fundamental parameter for chronic health risk assessments. It can be calculated by Equation (5):

EDI = (C × F_IR_ × TR)/(W_AB_ × 1000) (5)

where F_IR_ is the tea ingestion rate (g/person/day), C is the metal content (mg·kg^−1^), TR is the transference rate of the element from tea to tea infusion, and W_AB_ is the average body weight (60 kg for adults [31]). According to the report of Wu [32], the daily consumption of dry tea leaves was 11.4 g/person/day for adults.

#### 2.6.2. Risk of Individual Metals by Consuming Tea Leaves

The potential noncarcinogenic effects of individual metals can be quantitatively evaluated by the target hazard quotient (THQ), which can be calculated by using the Equation (6) [31,33]. A THQ value less than 1 indicates that there is no significant risk of noncarcinogenic effects for the exposed population. The occurring probability of noncarcinogenic effects occurring increases with an increasing THQ value:

THQ = EDI/RfD(6)

where the RfD is the oral reference dose (mg/(kg·d)), the EDI is the daily average exposure dose (mg/(kg·d)), and the THQ is the target hazard quotient. The RfD values of the studied elements were set to be (mg/kg bw/day) the following: 10.0 × 10^−1^ for Al; 10.0 × 10^−4^ for Cd; 3.6 × 10^−3^ for Pb; 3.0 × 10^−1^ for Zn; 4.0 × 10^−2^ for Cu; 3.1 × 10^−4^ for As; 2.0 × 10^−4^ for Hg; 2.0 × 10^−2^ for Ni; 1.4 × 10^−1^ for Mn; 15.0 × 10^−1^ for Cr(III) [1,16,34]. The RfD value for Cr was set as the value for Cr(III) due to the fact that Cr(VI) could be reduced to Cr(III) under acidic conditions in the stomach [35].

#### 2.6.3. Combined Risk of Multiple Metals by Consuming Tea Leaves

The overall potential risk effects may result from the exposure of more than one contaminant. The hazard index (HI) was used to estimate the total noncarcinogenic health hazard caused by exposure to multiple metals [33]. Given the potential noncarcinogenic risk posed by a variety of heavy metals, it is assumed that the toxic effects of heavy metals in tea are additive rather than synergistic or antagonistic to human health. Therefore, it is equal to the sum of the target hazard quotients of all studied contaminants and can be estimated by the following Equation (7) [33]:

HI = THQ_1_ + THQ_2_ + THQ_3_ + ··· + THQ_n_ (7)

where the THQ_n_ is the THQ value of an individual metal, and the HI is the hazard index of multiple metals. If the HI is less than one, the exposure dose is lower than the adverse reaction threshold, which does not have a carcinogenic risk. If the HI is more than one, the exposure dose is more than the adverse reaction threshold, and it is very likely that the heavy metals have negative effects on human health. When the HI value is more than 10.0, there is a chronic toxic effect on human health [36].

### 2.7. Statistical Analysis

The descriptive statistics (maximum value (Max), minimum value (Min), mean, standard deviation (SD), and coefficient of variation (C.V)) were summarized and calculated using Microsoft Excel 2016. The Kolmogorov-Smirnov test and Pearson’s correlation were performed by using the SPSS 20.0 software for Windows (SPSS Inc., Chicago, IL, USA).

## 3. Results and Discussion

### 3.1. Concentrations of Heavy Metals and Al in Soil

The descriptive statistics of the studied metals as well as pH of soil in the Puan tea area are presented in Table 1. The Kolmogorov-Smirnov method was used to test the concentrations of ten metal elements and pH values in all soil samples. The results showed that the p values (asymptotic significance values, bilateral) were all greater than the significant level (α = 0.05), indicating that the contents of ten elements and pH values in soils were all subject to the normal distribution. Compared with the background values of these elements in A layer soil (0–20 cm) of Guizhou Province [25], the average contents of Al, Hg, Cr, and Cu in tea garden soil were 1.85, 1.09, 1.26, and 4.10 times higher than corresponding background values of the A layer soil in Guizhou Province, respectively. Besides, for sample points, the percentages of heavy metal exceeding background value of A layer soil in Guizhou Province were 100% for Al, 53.8% for Hg, 30.8% for As, 92.3% for Cr, 15.4% for Ni, 100% for Cu, and 7.7% for Zn.

In addition, the average contents of heavy metals (Pb, Cd, Hg, As, Cr, Ni, Cu, and Zn) did not exceed the Grade II Environmental Quality Standard for soil in China (GB 15618-1995) (pH < 6.5) [37]. However, for Cr, Ni, and Cu, the phenomenon of single point exceeding the standard was observed, and the rates of exceeding the standard were 15.38%, 15.38%, and 7.69%, respectively (*n* = 13). These low ratios indicated that the environmental quality of tea garden soil in Puan was generally good. The C.V (%) decreased in the following order: Mn > Cd > As > Ni > Cu > Zn > Hg > Cr > Pb > Al, which indicated that the spatial variability of Mn in soil was the strongest among these 10 elements.

The heavy metals (As, Cd, Pb, and Zn) contents in soil were low in present study, comparing to important tea producing areas, such as Guiding (22.8 mg·kg^−1^ for As; 0.466 mg·kg^−1^ for Cd; 67.1 mg·kg^−1^ for Pb) [19], Duyun (23.8 mg·kg^−1^ for As; 0.259 mg·kg^−1^ for Cd; 28.1 mg·kg^−1^ for Pb) [18], Meitan (18.6 mg·kg^−1^ for As; 0.248 mg·kg^−1^ for Cd; 38.6 mg·kg^−1^ for Pb) [18], south Anhui Province, China (151.73 mg·kg^−1^ for Zn; 68.02 mg·kg^−1^ for As) [12], and north Iran (121.75 mg·kg^−1^ for Zn; 68.02 mg·kg^−1^ for As) [38]. On the contrary, the contents of Cu and Cr were high in study area, comparing to other tea producing areas, such as the Yaan area in Sichuan Province, China (33.42 mg·kg^−1^ for Cu; 88.9 mg·kg^−1^ for Cr) [39], Fujian Province, China (13.85 mg·kg^−1^ for Cu; 52.95 mg·kg^−1^ for Cr) [40], Jinghua in Zhejiang Province, southeast China (45.7 mg·kg^−1^ for Cu) [41], Wushwush farms, Ethiopia (10.51 mg·kg^−1^ for Cu) [10], and north Iran (13.01 mg·kg^−1^ for Cu) [38].

Soil pH ranged from 3.74 to 4.82, with an average value of 4.29 in the Puan tea area, reflecting an extreme acidity (Table 1). The research indicated that the application of nitrogen fertilizer may acidify the soil by the action of nitrobacteria within a week. Besides, manure includes organic and inorganic nitrogen, thus, organic nitrogen in manure may be slowly converted to nitrate, but inorganic nitrogen may be rapidly converted to nitrate, producing H^+^ [42]. Moreover, it was well-known that tea leaves are picked as raw materials, and nitrogen fertilizers need to be applied in order to ensure the tea yield. Therefore, the continual application of nitrogen fertilizers was the suspected main cause of soil acidification in the tea gardens. Furthermore, the geological setting of sandstone and shale and the characteristics of tea plant roots generating organic acids had a certain contribution to soil acidification in the tea gardens.

### 3.2. The Results of Soil Heavy Metal Pollution Evaluation

**Figure 2:**
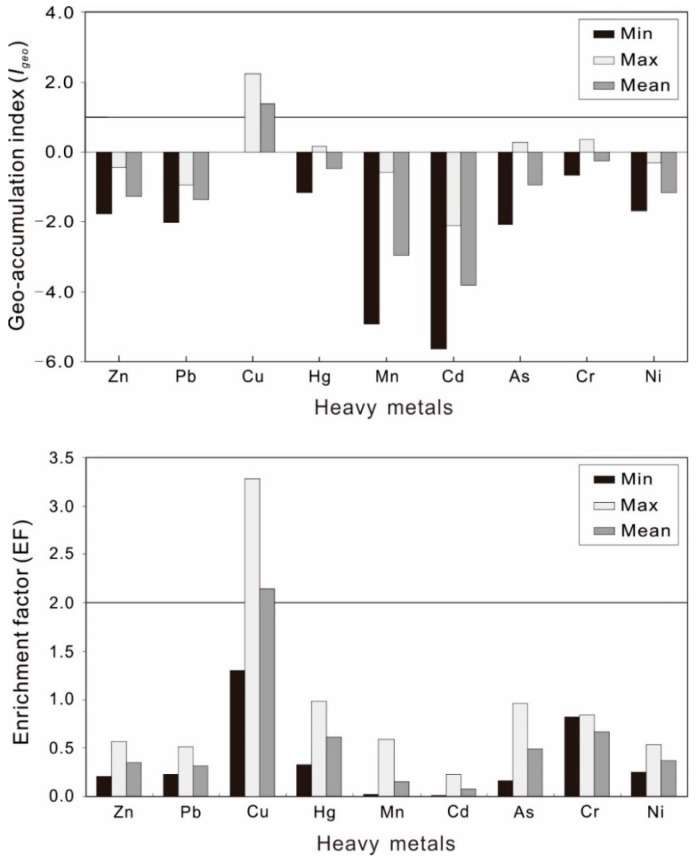
Maximum, minimum, and average values of geo-accumulation index and enrichment factor for the analyzed heavy metals in soils (0–30 cm) of Puan tea planting area, Guizhou Province, China.

To identify heavy metal pollution levels in soil, various methods have been proposed. In this study, the geo-accumulation index (*I_geo_*), enrichment factor (EF), and potential ecological risk index (RI) were applied to identify the levels of metal pollution in soil. The minimum, maximum, and average values of *I_geo_* and EF for different heavy metals in the surface soil were shown in Figure 2. Based on the *I_geo_*, the contamination of the investigated surface soil samples by Cu is characterized by the following criteria: uncontaminated to moderately contaminated, moderately to heavily polluted, and moderately contaminated based on the minimum (0.006), the maximum (2.222), and the average value (1.371), respectively. The average values of *I_geo_* for the other metals (Zn, Pb, Hg, Mn, Cd, As, Cr, and Ni) in soil were less than zero, indicating that the soils were not contaminated. However, for As, Cr and Hg, the maximum *I_geo_* values were 0.271, 0.355, and 0.173 in surface soils, respectively, suggesting that the soil was uncontaminated to moderately contaminated. In brief, based on the obtained average values of the geo-accumulation index (*I_geo_*), the order of heavy metals in surface soils, respectively, were as follows: Cu > Cr > Hg > As > Ni > Zn > Pb > Mn > Cd.

The EF has been proposed as a useful method for measuring heavy metal sources [21,26]. The descriptive statistical analysis of heavy metals EF values is presented in Figure 2. It was observed that the average EF values of individual heavy metals in the surface soil samples, respectively, decreased in the following order: Cu > Cr > Hg > As > Ni > Zn > Pb > Mn > Cd. Specifically, based on the average and maximum values of EF, the surface soil samples were found to present a moderate enrichment for Cu. On the contrary, the average EF values for other heavy metals (Zn, Pb, Hg, Mn, Cd, As, Cr, and Ni) were below two, indicating deficient to minimal enrichment in soil, whereas, the research showed that the contents of Cu in sandstone and shale of the upper Permian Longtan Formation were 143.2 mg·kg^−1^ and 200.0 mg·kg^−1^ near the tea area in Puan County [43].

Equally important, in the preliminary study, the average content of Cu in bedrock collected from the Puan tea growing area was 128.6 mg·kg^−1^ (unpublished data, *n* = 13). It was implied that the geochemical background value of Cu was high in the Puan tea growing area. In addition, the correlation coefficient of Cu and Al in soil was 0.728 (*p* < 0.01), showing a very significantly positive correlation, which also indicated that the main source of Cu in soil was the geological background in study area. Also, the content of Cu in soil was positively correlated with that of Ni in soil (r = 0.585, *p* < 0.05, *n* = 13), suggesting that the Cu content in soil was controlled by the geological setting in the studied area, because the Cr and Ni in cultivated soil are mainly from the natural geological background [44,45]. Consequently, the results of *I_geo_* and EF evaluation showed that the soil was moderately polluted by Cu in Puan tea planting area, which could be attributed to the high geochemistry background value of Cu in the regional strata. However, the average content of Cu in soil did not exceed the standard limited value (150 mg·kg^−1^) in the Grade II of Environmental Quality Standard for soil in China (GB15618-1995) (pH < 6.5) [37], indicating the soil environmental quality was good in Puan tea growing area, Guizhou Province, China.

The potential ecological risk indices ($E_{r}^{i}$) for each element were summarized in Table 2. The average $E_{r}^{i}$ values were in the following order: Hg > Cu > As > Cd > Ni > Pb > Cr > Zn > Mn. According to the categorizing basis for potential ecological risk index in the present study, Cu, As, Cd, Ni, Pb, Cr, Zn, and Mn would pose a low ecological risk to the environment, for they had the maximum $E_{r}^{i}$ values less than 40. In contrast, Hg posed a moderate ecological risk with an average $E_{r}^{i}$ value of 44.98 (higher than 40 and lower than 80). Equally, the highest value of $E_{r}^{i}$ for Hg was observed as 67.64, which was higher than 40 and less than 80, showing that Hg posed a moderate ecological risk at this site. Nevertheless, although the concentration of Cu in soil were high in the study area, the highest value of $E_{r}^{i}$ for Cu was 35 (less than 40), representing a low ecological risk because of the low toxicity response factor of Cu. Accordingly, the $E_{r}^{i}$ values of Hg and Cu were obviously greater than those of the other studied heavy metals. Thus, these two heavy metals represent priority pollutants in Puan tea planting area, Guizhou Province, China.

Based on risk index (RI) values for all heavy metals in this study, 12 sites had low ecological risks because the RI values of these sites were all lower than 110. However, only one site (PT12) had a value of 110 ≤ RI < 220, which would pose a moderate ecological risk to the environment. On the whole, all the soil samples in the study area have a low ecological risk according to the lower RI values with an average RI value of 88.22.

The potential ecological risk index (RI) result was similar to the results of the *I_geo_* and EF in essence, but there were differences for some elements. For example, the evaluation results of Hg based on the geo-accumulation index and enrichment factor index were categorized to unpolluted. However, Hg posed a moderate ecological risk to the soil environment according to the result of the ecological risk assessment, because the emphases of these three evaluation methods are different. To be specific, the *I_geo_* and EF can directly offer the heavy metal pollution level, but the biological toxicities of heavy metals were not taken into consideration. While, the potential ecological risk index makes up for the deficiencies of the *I_geo_* and EF, which is the effective complement to the *I_geo_* and EF, and reflects the toxicity and relative contribution proportion of heavy metals pollution to the organism. Therefore, in the assessment process of soil heavy metal pollution level and potential ecological risk level, it is necessary to combine these methods in order to evaluate soil heavy metal pollution comprehensively and scientifically.

The correlation coefficient of Cd and Pb in soil was 0.613 (*p* < 0.05, *n* = 13), which indicated that both Pb and Cd in soil have a source of traffic activity in the study area, because the traffic activity had a certain effect on the Pb and Cd contents in surrounding soil [46]. Furthermore, there are no mineral resource exploitation and smelting activities in the Puan tea growing area. As a result, the sources of heavy metals in the studied region may be mainly sources from the geology setting, transportation, and the application of pesticides and chemical fertilizers.

### 3.3. The Contents of Heavy Metals and Aluminum in Tea Leaves

The statistical results of concentrations of Al and 9 heavy metals (Mn, Pb, Cd, Hg, As, Cr, Ni, Cu, and Zn) in the tea leaves (young and mature) from the Puan tea area in Guizhou Province are shown in Table 3. The Kolmogorov-Smirnov method was used to test the concentrations of ten metal elements in tea leaves samples (young and mature). The results showed that the p values (asymptotic significance values, bilateral) were all greater than the significant level (α = 0.05), indicating that the contents of ten elements in tea leaves samples (young and mature) were all subject to the normal distribution. Compared with the national food safety standard (GB 2762-2017) [4] and the Ministry of Agriculture Tea heavy metals limited standards (NY/T 288-2012, NY 659-2003) [5,6], the concentrations of Pb, Cu, As, Hg, Cd, and Cr in young tea leaves and mature tea leaves were lower than the standard limit values (5.0, 30, 2.0, 0.3, 1.0, and 5.0 mg·kg^−1^ for Pb, Cu, As, Hg, Cd, and Cr, respectively) in the Puan tea area, showing that the quality of tea was good in Puan tea planting area. The average concentrations (mg·kg^−1^) of heavy metals and Al in young tea leaves decreased in the following order: Mn > Al > Zn > Cu > Ni > Cr > Pb > As > Hg > Cd (Table 3).

In the mature tea leaves, the other elements showed similar change characteristics with the young tea leaves, except for the contents of Al and Mn. Besides, the average contents of Al, Mn, Pb, Cd, Hg, As, and Cr in mature tea leaves were significantly higher than those in young tea leaves (*p* < 0.05). In particular, the difference of Al was the biggest, and the average content of Al in mature tea leaves (*n* = 13) was 20.4 times than that of Al in young tea leaves. According to a study, aluminium (Al) adversely affects plant growth because of toxicity. In particular, when ‘Riodeva’ (a rye genotype) was exposed 1.85 mM Al, Al toxicity signals, such as a severe decrease in root growth, occurred sooner, and the antioxidant responses were followed after 3 weeks in roots and leaves [47]. Likewise, several components of the cell in maize (*Zea mays* L.) may be harmed by oxidative stress (caused by Al), depending on the plant species. Moreover, in the sensitive maize line, Al^3+^ treatment and oxidative stress aroused cell death in root tip cells [48]. It was found that most of Al^3+^ in tea plant exist in chelate complexes or organic matter, which can be considered as one of the mechanism of Al-accumulating and Al-tolerance in tea plant [49]. The amount of aluminum can promote the growth of tea plant, which is beneficial to the stability of the root cell membrane of tea plant, and promote the absorption and accumulation of Al in tea plant [50,51]. The contents of Mn in tea leaves were very high, and the average content of Mn in mature tea leaves was 1940 mg·kg^−1^, while the average content of Mn in young tea leaves was 556 mg·kg^−1^. The results of numerous studies show that the contents of Mn and Al in tea were very high [12,13,16,52,53]. The average content of Pb (0.931 mg·kg^−1^) in mature tea leaves was 4.9 times than the content of Pb (0.190 mg·kg^−1^) in young tea leaves. The content of Cu in young tea leaves was significantly higher than that in mature tea leaves (*p* < 0.05), The content of Cu in young tea leaves was high in the Puan tea area, and the Cu contents ranged from 14.90 to 26.10 mg·kg^−1^ with an average concentration of 18.57 mg·kg^−1^, which was higher than the average Cu content (16.2 mg·kg^−1^) of 24 brands of tea obtained from five provinces (Zhejiang, Henan, Fujian, Guangdong, and Yunnan) in China [54]. This may be related to the higher background value of Cu content in soil and rock collected from the present research area. The average content of Zn in young tea leaves (42.2 mg·kg^−1^) was significantly higher than that in mature tea leaves (*p* < 0.05), and it was 3.1 times as much as that of mature tea leaves (13.5 mg·kg^−1^). Also, the Zn content of young tea leaves was higher than the standard (40.0 mg·kg^−1^) of Zn-enriched tea recommended by General Administration of Quality Supervision, Inspection and Quarantine of the People’s Republic of China (AQSIQPRC) [55], which illustrated that the tea is very suitable to develop Zn-enriched tea in the Puan tea region. The contents of Cu and Zn in young leaves of tea plant were higher, which may be related to the vigorous metabolism of young leaves of tea plant. As the main trace nutrient elements, Zn and Cu play an importance role in the process of tea plant metabolism and are some portions of certain substances in plant components (such as CuZnSOD, anti-aging and promoting the synthesis of auxin, such as indoleacetic acid). In addition, Cu and Zn may have antagonistic actions on other heavy metals, resulting in the relative enrichment of Zn and Cu in young tea leaves, while other heavy metals were relatively enriched in mature tea leaves. Nevertheless, there was no significant difference for the contents of Ni in young tea leaves and mature tea leaves. The average BCF values of heavy metals and Al in tea leaves from the Puan tea garden area in Guizhou are shown in Table 3. It can be seen that the accumulation ability of tea to Mn was the strongest among the ten elements. Similarly, Ma et al. [56] and Fang et al. [12] also found that tea leaves had a strong accumulation ability for Mn. For instance, Fang et al. [12] found that the BCF values of Mn in mature tea leaves and young tea leaves of typical tea plantations were 5.3 and 1.6, respectively, in South Anhui Province, China. The BCF values of heavy metals and Al in young tea leaves decreased in the following order: Mn > Cd > Zn > Hg > Ni > Cu > As ≈ Pb > Cr > Al (Table 3), while the BCF values of heavy metals and Al in mature tea leaves decreased in the following order: Mn > Cd > Hg > Ni > Zn > Cu > Al > Pb > As > Cr (Table 3). In particular, the BCF values of Mn and Cd in the young and mature tea leaves were higher, which may be related to the acidic soil of the tea garden (pH = 4.29, *n* = 13), because in acidic soil, the migration of Mn and Cd are stronger. A higher BCF value indicates a higher transfer potential of metals in tea leaves, Hence, the above results indicated that Mn had a higher capacity of absorption into tea leaves from soil than the other heavy metals studied. In general, the bioavailability and toxicity of metals in soil are significantly influenced by pH conditions. It was reported that the water-soluble Mn content in soil increased abruptly with decreasing pH in the region when pH was below 4.5 [42]. The mechanism for this phenomenon can attribute to an increase in solubility and ion competition. As soil pH decreases, concentrations of Mn^2+^, Zn^2+^, Cu^2+^, and Cd^2+^ increases in soil solution. This enhances the competition of free ions and reduces the adsorption to soil particles. The high BCF values of Mn and Cd indicated that Mn and Cd were more easily absorbed by tea plants than other elements. Moreover, Mn in soils is typically present in the form of pyrochroite (Mn(OH)_2_), hausmannite (Mn_3_O_4_), manganite (γ-MnOOH), birnessite (δ-MnO_2_), and a freshly precipitated form of MnCO_3_ [42]. The chemical forms of Mn present in soil are known to depend on soil pH. Valence electrons in Mn_3_O_4_ and γ-MnOOH can rearrange themselves spontaneously to give δ-MnO_2_ and Mn^2+^ in an acid soil by the following two reactions:

Mn_3_O_4_ + 4H^+^ → δ-MnO_2_ + 2Mn^2+^ + 2H_2_O

4MnOOH + 4H^+^ ⇌ 2MnO_2_ + 2Mn^2+^ + 4H_2_O

Accordingly, Mn^2+^ which is available for tea plants is released from soil by H^+^, which is produced from NH_4_^+^ (the application of nitrogen fertilizer) [10,42]. On the other hand, the correlation analysis of pH values and heavy metal accumulation is commonly used in some researches on bioconcentration of heavy metal from soil to plants owing to the close relation between soil pH and heavy metal properties. In addition, heavy metal uptake by food crops depends upon soil physico-chemical characteristics and plant species [57]. Furthermore, although the concentrations of Al in tea leaves were higher, the BCF values of Al in young tea leaves and mature tea leaves were lower, which was due to the fact that Al was a major element in soil and its average content (106 g·kg^−1^) was significantly higher than those of other elements. To sum up, soil pH is considered to be one of the most important factors influencing the transfer of metals from soil to plants, and higher pH values have been found to reduce the bioavailability and toxicity of some metals. Therefore, it was advocated that some suitable dolomite powders and organic fertilizers were applied to the acidulated soil for improving the extremely acid soil in tea garden.

### 3.4. Correlation between Metal Elements in Tea Leaves and Soil Physico-Chemical Properties

The correlation between the contents of heavy metals and Al in tea leaves (young and mature) and the contents of corresponding elements and pH in soil was analyzed, and the results are shown in Table 4. In particular, the contents of Mn, Al, Cu, Ni, As and Hg in young leaves were positively correlated with the contents of corresponding elements in soil, but not significantly, indicating that the soil was not the only source of these elements in young tea leaves, and there may be other sources of these metals in young leaves. In contrast, the contents of Pb, Cd, Cr, and Zn in young tea leaves were negatively correlated with the contents of corresponding elements in soil. Especially, Pb reached a significant level (*p* < 0.05), indicating that the Pb in soil will inhibit the absorption of Pb by young leaves when the concentration of Pb was too high. Therefore, the contents of heavy metals in the young tea leaves were affected by not only the element content in soil, but also the heavy metal fractionations in soil, atmospheric conditions, precipitation and the migration of metals in the system of soil-root-stem-branch-leaf. Results from a study conducted by Jin et al. [9] indicated that atmospheric dry deposition was one of the important reasons leading to heavy metal pollution in tea leaves.

The contents of Mn, Pb, Cd, and As in the mature leaves were positively correlated with the contents of the corresponding elements in soil, while and the contents of Cu, Zn, Hg, Cr and Ni in the mature leaves were negatively correlated with the contents of the corresponding elements in soil, but none of them reached a significant level. Therefore, the metal elements in soil were not the only source of metal elements in mature leaves, and there may be other sources, such as atmospheric dry deposition. In addition, the correlation between the contents of metal elements in soil and corresponding tea leaves (young and mature) was quite different, and these results showed that there were differences in the accumulation ability of young tea leaves and mature tea leaves to metal elements. Wu et al. [58] conducted that the biological characteristics of young leaves are different from those of mature leaves, and there were different accumulation abilities of metal elements in different parts of tea plant. Bisides, the concentrations of Al, Mn, Pb, Cd, Hg, As, Cr, Ni, and Zn of mature leaves were negatively correlated with soil pH, and Cd reached a significant level (*p* < 0.05), indicating that heavy metals and Al easily migrated to mature tea leaves and were accumulated when the soil was acidic. Moreover, the contents of Mn and Al in young leaves and mature leaves were negatively correlated with soil pH, which indicated that soil pH had a greater effect on the contents of Mn and Al in tea leaves and it might be the key factor of controlling the enrichment Mn and Al in mature tea leaves. Similar conclusions had been drawn by Fang et al. [12] and Xie et al. [59]. Furthermore, the concentrations of Mg, Al, Fe, Mn, Cr, Cu, Zn, As, Pb, and Ni in young tea leaves and mature tea leaves were not obviously correlated with soil organic matter (SOM) (*p* > 0.05), indicating that soil organic matter had no effect on the contents of heavy metals in tea leaves [12]. However, Zhang and Fang [41] reported that both acidification and accumulation of dissolved organic matter (DOM) induced by tea plantations were also important causes of increased accumulation of the metals in tea leaves. Therefore, the influence of soil organic matter on heavy metals contents in tea leaves will need to be studied further in the Puan tea gardens in Guizhou Province, China.

### 3.5. Health Risk Assessment

#### 3.5.1. Estimated Daily Intakes of Al and Heavy Metals by Consuming Tea Leaves

The metal elements in tea leaves are not all transferred into the human body but rather leached into the tea infusion, and then transferred into the human body. Therefore, the transference rates (TR) of metal elements from tea to tea infusion need to be considered when calculating the amount of metal elements in tea leaves into human body. In present study, the determination of the concentrations of metal elements in tea infusion had not been carried out, and the transference rates of metals from tea to tea infusion were quoted from the results of some previous studies. Specifically, the transference rates of Cu, Zn, As, Mn, Al, and Cr were 28.7%, 19.3%, 16.2%, 22.5%, 20.2%, and 42%, respectively [60]. The transference rates of Cd, Pb, Hg, and Ni were 6.6%, 19.8% [1], 45.2% [19], and 30% [16], respectively.

The estimated daily intakes (EDI) values of metals through the consumption of tea leaves from Puan tea area were calculated and are summarized in Table 5. The health risk assessment was only performed for adults, since the children rarely have the habit of drinking tea. For local adults, the decreasing estimated daily intakes (EDI) values of the studied metals in young tea leaves was the following: Mn > Al > Zn > Cu > Ni > Cr > Pb > As > Hg > Cd, indicating that the contribution of Mn was the highest among the daily intakes of heavy metals. Similarly, Gruszecka-Kosowska et al. [16] showed that the estimated daily intake (EDI) of Mn was the highest among the 16 metals (Mn, Al, Fe, Zn, Cu, Se, Sn, Ni, Pb, Sb, Mo, Tl, Co., Cr, Hg, and Cd) through the consumption of tea leaves. The EDI value for Al in young tea leaves in this study was similar to those in green tea infusions (6.50 × 10^−2^ mg/kg bw/day) [1] and Puerh tea infusions (0.41 mg/kg bw/day) [11]. However, For Pb, the EDI value for young tea leaves in present study was about 100 times less than the value (1.23 × 10^−4^ mg/kg bw/day) reported by Li et al. [1]. Also, in present study, for As, the EDI value for young tea leaves was about 100 folds lower than the value (2.67 × 10^−4^ mg/kg bw/day) reported by Nkansah et al. [17]. In addition, for Cu and Zn, the EDI values of the young leaves in present study were higher than those of the mature tea leaves, indicating that young tea leaves were more beneficial to supplement of Zn and Cu than mature tea leaves. To be specific, it was caused by the concentrations of Cu and Zn in young tea leaves which were higher than those in mature tea leaves. On the contrary, the mature leaves were higher than the young leaves for the EDI values of the other eight kinds of metal elements (Al, Mn, Pb, Cd, Hg, As, Cr, and Ni), implying that drinking mature tea infusions could increase the source of heavy metals in human body. The EDI values in this study were all below the RfD values for all the ten elements in young tea leaves, suggesting that the consumption of the studied young tea leaves did not seem to pose a health risk to local adults. Otherwise, for mature tea leaves, the EDI values of all the elements were lower than the corresponding RfD values, except for Mn of the PLY3 sample.

#### 3.5.2. Risk of Individual Metal by Consuming Tea Leaves

The THQ, proposed by US EPA [33], has been successfully used to qualitatively assess the possible non-carcinogenic effects of heavy metals on humans via food consumption [1,31,61]. As shown in Table 6. The THQ values of individual metals through the ingestion of young tea infusions were all below one, suggesting that the daily intake of individual metals via the consumption of young tea leaves would be unlikely to result in adverse health effects for adults, which was consistent with the reports of Cao et al. [11], Shen and Chen [61] and Li et al. [1]. For young tea leaves, the trend of THQ values was in the order of Mn > Ni > Cu > Hg > As > Al > Zn > Pb > Cd > Cr. Similarly, Gruszecka-Kosowska et al. [16] have studied the health risk derived from oolong tea drinking for Polish consumers and found that the THQ values of individual metals were all below one and in the order of Mn > Al > Zn > Cd > Cr. In contrast, the THQ values of individual metals via the consumption of both wheat grain and leaf vegetables decreased in the orders of Cr > Pb > Hg > Ni [62] and Cr > Cd > Pb > Hg [63].

The opposite trends of the THQ values may be related to the internal difference of various plants accumulating metals from surrounding environments [1]. For mature leaves, the average THQ values of each metal decreased in the order of Mn > Al > As > Hg > Ni > Cu > Pb > Zn > Cd > Cr. The average THQ value of Mn in the mature leaves was the highest, with an average value of 5.79 × 10^−1^ mg/kg bw/day. Especially, the THQ value of Mn in PLY3 was higher than one, and it could have a certain health risk, which was related to the strong accumulation ability of Mn in mature tea leaves. The relatively high THQ value of Mn may be attributed to its high concentrations in tea leaves (556 mg·kg^−1^ for young leaves and 1940 mg·kg^−1^ for mature leaves). The results of the present study indicated that the health risk could exist via drinking mature tea infusions because of the high content of Mn in mature tea leaves. Nevertheless, the bioavailability (% of intestinal absorption) of Mn is 8% [64], thus, less Mn is actually absorbed by the human body and the information allow any health risk for adults to be deserted. However, further toxicological experiments on Mn should be performed.

#### 3.5.3. Combined Risk of Multiple Metals by Consuming Tea Leaves

The combined non-carcinogenic effects of multiple metals can be expressed by their HI values. The HI values of all studied metals via the consumption of young and mature tea infusions are listed in Table 6. It was found that HI values for all the studied metals, in the young tea leaves, were below 1, which allowed us to conclude that there is essentially no carcinogenic risk from the consumption of the young tea infusions from the Puan tea area, in the quantities and with the frequency assumed in the present study. On the contrary, there might be potential health risks through the consumption of the mature tea infusions, with five out of 13 samples having HI values above one in this region. In particular, the relative contribution ratio of Mn to HI values in this study ranged from 54.9% to 79.3% for the five samples with HI values higher than one, showing manganese was the major element in contributing to the potential health risk through consuming mature tea infusions. Thus, more work should be conducted on monitoring Mn contents in tea leaves. Moreover, it was reported that 95.0% Mn existed in the form of Mn(II) and the remaining 5.0% existed in the form of total organic-bound Mn in tea infusions. In the later case, manganese is rather poorly absorbed from the small intestine by a mechanism similar to the absorption of iron, and it might be suggested that the bioavailability of manganese in tea infusions was lower for adults [52]. The quality of tea made of old leaves and branches was considered terrible, especially by certain tribes in China, because of the higher contents of Mn and Al [54]. Likewise, ‘available’ manganese from Tetley tea bag samples accounted for 115.5% of the average daily dietary intake of Mn, which was of some concern, as high manganese in the diet can lead to long-term toxicity [53]. Although the average HI value of mature tea leaves was less than one (0.97), HI values above one existed for some mature tea leaves samples. In detail, the percentage of samples of HI values above one was 38.46%. Despite all this, through drinking mature tea infusion from Puan county, the health risks of heavy metals and Al could be not worth mentioning as a result of the low bioavailability of manganese and high contribution ratio of manganese to health risk [64]. Furthermore, the absorption of Mn in the gastrointestinal tract is low, and it has a two-way regulation mechanism. Namely, when Mn is lacking, the absorption rate rises; when Mn is sufficient, the absorption rate is reduced [65]. Additionally, a study indicated that the hazard of excessive element intake through black tea consumption should be considered as inappreciable in Iran, however, the risk related to manganese appeared to be more than other toxic metals [66]. Therefore, when making use of mature tea leaves to develop other tea products, the excessive Mn intake should be prevented, and some measures should be implemented to control excessive manganese consumption. Simultaneously, further studies will be needed to conduct to confirm this finding and its related consequences. The effects of Mn and Al on human health should be identified urgently by in-depth studies.

In the present health risk assessment, there were some uncertain factors. For example, the sample quantity was limited, and its representativeness was relatively circumscribed. Besides, only the risks from 10 metal elements (Mn, Al, As, Hg, Ni, Cu, Pb, Zn, Cd, and Cr) were taken into consideration through drinking tea, without considering the influence of other exposure ways on the health risk, such as food, breathing, skin contact and other harmful elements (such as antimony, tin, cobalt, thallium, iron, and rare earth element), which underestimated the risk of heavy metals. On the contrary, in the calculation of exposure dose, the occurrence forms of metal elements were not considered, and the total exposure dose was calculated directly, so that the evaluation results were higher. Moreover, in this study, a questionnaire survey of the local human was not carried out, and the exposure parameters were mainly quoted from some previous research results. Furthermore, in the calculation of daily intake, the same transference rates were quoted for the same metal elements in young leaves and mature leaves. Therefore, there may be some deviations between the final health risk value and the real valued in the present study.

## 4. Conclusions

The average contents of heavy metals in soil from the Puan tea growing area in Guizhou Province, China were below the Grade II of Environmental Quality Standard for soil in China (GB 15618-1995, pH < 6.5) [37]. The geo-accumulation index and enrichment factor showed that the Cu in soil was enriched moderately, and the background value of Cu in soil was high. Besides, the concentrations of all studied metals in tea leaves satisfied their relevant recommended maximum limits of current standards for tea leaves (NY/T 288-2012, NY 659-2003, and GB 2762-2017) [4,5,6], and the quality of tea leaves in Puan was good. In addition, the contents of Cu and Zn in young tea leaves were significantly higher than that in mature tea leaves (*p* < 0.05), while the average contents of Al, Mn, Pb, Cd, Hg, As, and Cr in mature tea leaves were significantly higher than these in young leaves (*p* < 0.05). The content of Mn in tea was very high, the average contents of Mn in mature tea leaves and young tea leaves were 1940 mg·kg^−1^ and 556 mg·kg^−1^, respectively. Moreover, the BCFs of heavy metals and Al in young tea leaves decreased in the following order: Mn > Cd > Zn > Hg > Ni > Cu > As ≈ Pb > Cr > Al. Specifically, the accumulation ability of tea to Mn is the strongest, and the BCF of Mn in mature tea leaves was 12.5. Mn, Al, Cu in young leaves were positively correlated with soil corresponding element contents, and Pb in young leaves and Pb in soil have significantly negative correlation (*p* < 0.01). Al, Mn, Pb, Cd, Cr, Ni, Zn in mature leaves were negatively correlated with the soil pH. In particular, Cd reached a significant level (*p* < 0.05). Furthermore, the average THQ and HI values of young tea leaves were less than one, indicating that the consumption of young tea infusions would not result in health risks of heavy metals to adults. For mature tea leaves, although the percentage of HI values above one was 38.46%, the health risks of heavy metals and Al could be negligible as a result of low bioavailability of manganese through drinking mature tea infusion in Puan County (Guizhou Province, China). Nevertheless, it was suggested that as a precaution excessive Mn intake should be prevented when making use of mature tea leaves to develop other tea products.

## Figures and Tables

**Table 1:** Descriptive statistics of heavy metal concentrations (mg·kg^−1^) in soil (*n* = 13). ^a^ Standard deviation; ^b^ Coefficients of variation (%); ^c^ data was collected from Wei et al. [25]; ^d^ Grade II of Environmental Quality Standard for soil in China (GB 15618-1995) [37].

Sample ID	pH	Al	Mn	Pb	Cd	Hg	As	Cr	Ni	Cu	Zn
PT1	4.82	115,000	39	17.8	0.02	0.07	14.7	111	19.6	137.5	44
PT2	4.22	65,200	109	12.9	0.02	0.13	21.7	111	18.5	48.2	55
PT3	4.34	109,500	394	21.9	0.03	0.16	24.6	107	37.5	115.5	109
PT4	4.16	108,000	109	23.9	0.05	0.15	28.9	108	19.9	91.6	62
PT5	4.22	12,050	240	22.4	0.07	0.14	15.8	91	43.8	224.0	78
PT6	4.16	106,000	197	23.5	0.15	0.09	14.0	108	22.2	147.5	62
PT7	4.15	111,000	95	22.7	0.07	0.10	12.7	184	22.0	118.5	46
PT8	4.09	95,900	794	22.3	0.11	0.14	13.1	143	26.8	145.5	74
PT9	4.38	111,000	245	19.5	0.11	0.10	8.6	109	32.7	148.0	76
PT10	4.73	122,000	220	18.2	0.12	0.10	7.1	138	30.1	145.5	64
PT11	4.29	106,000	68	20.4	0.12	0.11	36.2	103	18.1	144.0	54
PT12	4.52	128,500	53	18.2	0.06	0.19	10.4	155	47.8	149.5	48
PT13	3.74	85,000	215	27.4	0.23	0.13	19.1	101	23.0	90.3	62
Max	4.82	128,500	794	27.4	0.23	0.19	36.2	184	47.8	224.0	109
Min	3.74	65,200	39	12.9	0.02	0.07	7.1	91	18.1	48.2	44
mean	4.29	106,400	214	20.9	0.09	0.12	17.5	121	27.8	131.2	64
SD ^a^	0.32	16,646	201	3.6	0.06	0.03	8.4	26	9.9	41.5	17
C.V (%) ^b^	7.4	15.6	94.1	17.3	66.7	25.4	48.3	21.9	35.6	31.6	27.1
background values of A layer soil in Guizhou province ^c^	6.20	57,600	794	35.2	0.659	0.110	20.0	95.9	39.1	32.0	99.5
the number of samples with higher than Background values	0	13	0	0	0	7	4	12	2	13	1
limit values (pH < 6.5) ^d^		-	-	250.0	0.3	0.300	40.0	150	40.0	150.0	200
the number of samples with higher than limit values		-	-	0	0	0	0	2	2	1	0

**Table 2:** Minimum, maximum, and mean of single ecological risk ($E_{r}^{i}$) and potential ecological risk index (RI) in surface (0–30 cm) soils in the Puan tea region, Guizhou Province, China.

Sample ID	$E_{r}^{i}$									RI
	Zn	Pb	Cu	Hg	Mn	Cd	As	Cr	Ni	
PT1	0.44	2.53	21.48	26.91	0.05	0.91	7.35	2.31	2.51	64.49
PT2	0.55	1.83	7.53	45.45	0.14	0.91	10.85	2.3	2.37	71.95
PT3	1.10	3.11	18.05	57.45	0.50	1.37	12.30	2.23	4.80	100.90
PT4	0.62	3.39	14.31	56.00	0.14	2.28	14.45	2.25	2.54	95.99
PT5	0.78	3.18	35.00	50.18	0.30	3.19	7.90	1.90	5.60	108.04
PT6	0.62	3.34	23.05	32.73	0.25	6.83	7.00	2.25	2.84	78.90
PT7	0.46	3.22	18.52	36.36	0.12	3.19	6.35	3.84	2.81	74.87
PT8	0.74	3.17	22.73	51.64	1.00	5.01	6.55	2.98	3.43	97.25
PT9	0.76	2.77	23.13	37.82	0.31	5.01	4.30	2.27	4.18	80.55
PT10	0.64	2.59	22.73	35.64	0.28	5.46	3.55	2.88	3.85	77.62
PT11	0.54	2.90	22.50	41.45	0.09	5.46	18.10	2.15	2.31	95.51
PT12	0.48	2.59	23.36	67.64	0.07	2.73	5.20	3.23	6.11	111.41
PT13	0.62	3.89	14.11	45.45	0.27	10.47	9.55	2.11	2.94	89.42
Max	1.10	3.89	35.00	67.64	1.00	10.47	18.10	3.84	6.11	111.41
Min	0.44	1.83	7.53	26.91	0.05	0.91	3.55	1.90	2.31	64.49
Mean	0.64	2.96	20.50	44.98	0.27	4.06	8.73	2.52	3.56	88.22
Contribution rate (%)	0.73	3.36	23.24	50.99	0.31	4.60	9.90	2.86	4.04	100

**Table 3:** Concentration (mg·kg^−1^) of heavy metals and Al in young and mature tea leaves collected from Puan tea planting area, Guizhou Province, China (*n* = 13).

Tea Leaves Type	Descriptive Statistics	Al	Mn	Pb	Cd	Hg	As	Cr	Ni	Cu	Zn
Young tea leaves (*n* = 13)	Max	660	1130	0.400	0.092	0.085	0.456	1.26	14.90	26.10	50.3
	Min	250	194	0.107	0.012	0.014	0.073	0.33	6.33	14.90	35.8
	Mean	358	556	0.190	0.039	0.043	0.142	0.62	9.21	18.57	42.2
	standard deviation (±SD)	110	307	0.072	0.020	0.020	0.104	0.33	2.47	3.45	4.6
	coefficient of variation (C.V) (%)	30.6	55.2	38.0	50.7	47.2	73.1	52.6	26.8	18.6	10.8
	BCF	0.003	3.9	0.010	0.728	0.372	0.010	0.005	0.36	0.16	0.7
Mature tea leaves (*n* = 13)	Max	10,400	4610	1.265	0.087	0.089	0.453	2.91	14.20	16.25	20.0
	Min	4300	536	0.560	0.040	0.043	0.189	0.69	3.43	6.17	9.1
	Mean	7323	1940	0.931	0.061	0.063	0.291	1.47	9.44	10.70	13.5
	standard deviation (±SD)	1752	1120	0.196	0.012	0.015	0.067	0.70	3.55	3.42	2.6
	coefficient of variation (C.V) (%)	23.9	57.7	21.1	20.2	24.0	23.0	48.0	37.6	32.0	19.1
	BCF	0.07	12.5	0.046	1.176	0.550	0.020	0.013	0.38	0.10	0.2
limit values of safety standards ^a^		-	-	5.0	1.0	0.3	2.0	5.0	-	30.0	-

^a^ GB 2762-2017 [4], NY/T 288-2012 [5] and NY 659-2003 [6].

**Table 4:** Pearson’s correlation between metal elements in tea leaves and soil physico-chemical properties (*n* = 13).

Soil Properties	Tea Leaves Type	Al	Mn	Pb	Cd	Hg	As	Cr	Ni	Cu	Zn
Metal elements	Young tea leaves	0.347	0.504	−0.823 **	−0.124	0.072	0.075	−0.007	0.113	0.292	−0.298
	Mature tea leaves	0.063	0.457	0.330	0.290	−0.270	0.244	−0.416	−0.285	−0.017	−0.194
pH	Young tea leaves	−0.040	−0.178	0.269	0.020	0.020	0.179	0.239	0.149	0.250	0.148
	Mature tea leaves	−0.352	−0.275	−0.202	−0.570 *	−0.370	−0.459	−0.230	−0.156	0.116	−0.276

Note: ** Correlation is significant at the 0.01 level (2-tailed); * Correlation is significant at the 0.05 level (2-tailed).

**Table 5:** Estimated daily intakes (EDI) (mg/kg bw/day) of Al and heavy metals for adults due to the consumption of tea leaves in Puan tea growing area.

Sample ID	Estimated Daily Intakes (EDI)									
	Al	Mn	Pb	Cd	Hg	As	Cr	Ni	Cu	Zn
PNY1	9.60 × 10^−3^	8.09 × 10^−3^	8.80 × 10^−6^	5.64 × 10^−7^	5.32 × 10^−6^	3.20 × 10^−6^	3.75 × 10^−5^	3.61 × 10^−4^	1.31 × 10^−3^	1.51 × 10^−3^
PNY2	1.11 × 10^−2^	3.62 × 10^−2^	1.50 × 10^−5^	4.39 × 10^−7^	5.24 × 10^−6^	1.40 × 10^−5^	1.01 × 10^−4^	4.98 × 10^−4^	1.17 × 10^−3^	1.57 × 10^−3^
PNY3	1.07 × 10^−2^	4.72 × 10^−2^	7.79 × 10^−6^	3.89 × 10^−7^	2.75 × 10^−6^	2.52 × 10^−6^	3.11 × 10^−5^	4.22 × 10^−4^	1.03 × 10^−3^	1.47 × 10^−3^
PNY4	1.27 × 10^−2^	1.23 × 10^−2^	5.72 × 10^−6^	3.51 × 10^−7^	3.52 × 10^−6^	3.14 × 10^−6^	3.83 × 10^−5^	4.59 × 10^−4^	9.52 × 10^−4^	1.45 × 10^−3^
PNY5	2.53 × 10^−2^	2.24 × 10^−2^	6.40 × 10^−6^	1.15 × 10^−7^	3.01 × 10^−6^	4.56 × 10^−6^	3.91 × 10^−5^	4.52 × 10^−4^	1.42 × 10^−3^	1.84 × 10^−3^
PNY6	1.04 × 10^−2^	3.46 × 10^−2^	5.34 × 10^−6^	2.63 × 10^−7^	2.75 × 10^−6^	2.34 × 10^−6^	4.95 × 10^−5^	6.70 × 10^−4^	8.78 × 10^−4^	1.59 × 10^−3^
PNY7	1.54 × 10^−2^	9.36 × 10^−3^	6.40 × 10^−6^	5.64 × 10^−7^	1.20 × 10^−6^	2.62 × 10^−6^	3.19 × 10^−5^	4.04 × 10^−4^	8.42 × 10^−4^	1.76 × 10^−3^
PNY8	1.54 × 10^−2^	3.56 × 10^−2^	4.03 × 10^−6^	4.89 × 10^−7^	1.20 × 10^−6^	2.37 × 10^−6^	2.71 × 10^−5^	4.32 × 10^−4^	9.52 × 10^−4^	1.36 × 10^−3^
PNY9	1.73 × 10^−2^	1.67 × 10^−2^	7.82 × 10^−6^	7.27 × 10^−7^	4.04 × 10^−6^	3.14 × 10^−6^	9.58 × 10^−5^	8.49 × 10^−4^	9.92 × 10^−4^	1.38 × 10^−3^
PNY10	1.50 × 10^−2^	2.08 × 10^−2^	6.13 × 10^−6^	4.64 × 10^−7^	3.44 × 10^−6^	6.49 × 10^−6^	4.39 × 10^−5^	5.99 × 10^−4^	8.12 × 10^−4^	1.45 × 10^−3^
PNY11	1.34 × 10^−2^	3.39 × 10^−2^	7.22 × 10^−6^	3.76 × 10^−7^	3.01 × 10^−6^	4.71 × 10^−6^	4.07 × 10^−5^	6.73 × 10^−4^	8.34 × 10^−4^	1.69 × 10^−3^
PNY12	1.23 × 10^−2^	1.08 × 10^−2^	7.26 × 10^−6^	1.50 × 10^−7^	5.58 × 10^−6^	5.54 × 10^−6^	8.62 × 10^−5^	5.81 × 10^−4^	1.05 × 10^−3^	1.73 × 10^−3^
PNY13	1.04 × 10^−2^	1.40 × 10^−2^	4.85 × 10^−6^	3.89 × 10^−7^	7.30 × 10^−6^	2.25 × 10^−6^	2.63 × 10^−5^	4.23 × 10^−4^	9.08 × 10^−3^	1.31 × 10^−3^
Mean	1.38 × 10^−2^	2.32 × 10^−2^	7.14 × 10^−6^	4.86 × 10^−7^	3.72 × 10^−6^	4.38 × 10^−6^	4.98 × 10^−5^	5.25 × 10^−4^	1.01 × 10^−3^	1.55 × 10^−3^
PLY1	1.65 × 10^−1^	2.24 × 10^−2^	3.30 × 10^−5^	7.77 × 10^−7^	3.86 × 10^−6^	7.20 × 10^−6^	1.75 × 10^−4^	5.54 × 10^−4^	8.86 × 10^−4^	5.21 × 10^−4^
PLY2	2.23 × 10^−1^	1.27 × 10^−1^	3.20 × 10^−5^	9.03 × 10^−7^	4.47 × 10^−6^	9.02 × 10^−6^	1.43 × 10^−4^	8.09 × 10^−4^	7.03 × 10^−4^	4.66 × 10^−4^
PLY3	2.61 × 10^−1^	**1.93 × 10^−1^**	3.18 × 10^−5^	8.40 × 10^−7^	4.04 × 10^−6^	1.11 × 10^−6^	5.91 × 10^−5^	3.56 × 10^−4^	5.51 × 10^−4^	4.51 × 10^−4^
PLY4	3.26 × 10^−1^	5.02 × 10^−2^	4.76 × 10^−5^	7.02 × 10^−7^	6.01 × 10^−6^	8.74 × 10^−6^	9.10 × 10^−5^	6.38 × 10^−4^	7.85 × 10^−4^	5.17 × 10^−4^
PLY5	2.69 × 10^−1^	6.10 × 10^−2^	4.46 × 10^−5^	7.52 × 10^−7^	5.84 × 10^−6^	1.02 × 10^−5^	1.59 × 10^−4^	5.20 × 10^−4^	8.83 × 10^−4^	5.68 × 10^−4^
PLY6	2.26 × 10^−1^	1.16 × 10^−1^	3.62 × 10^−5^	5.89 × 10^−7^	6.36 × 10^−6^	8.25 × 10^−6^	9.18 × 10^−5^	3.15 × 10^−4^	3.70 × 10^−4^	4.00 × 10^−4^
PLY7	3.68 × 10^−1^	3.24 × 10^−2^	3.19 × 10^−5^	7.40 × 10^−7^	7.64 × 10^−6^	9.45 × 10^−6^	9.42 × 10^−5^	7.58 × 10^−4^	6.76 × 10^−4^	7.33 × 10^−4^
PLY8	2.26 × 10^−1^	9.66 × 10^−2^	2.11 × 10^−5^	8.90 × 10^−7^	3.69 × 10^−6^	5.82 × 10^−6^	8.46 × 10^−5^	4.33 × 10^−4^	4.22 × 10^−4^	4.73 × 10^−4^
PLY9	3.99 × 10^−1^	8.74 × 10^−2^	2.61 × 10^−5^	7.27 × 10^−7^	7.39 × 10^−6^	6.59 × 10^−6^	1.87 × 10^−4^	6.98 × 10^−4^	4.27 × 10^−4^	4.69 × 10^−4^
PLY10	3.07 × 10^−1^	7.40 × 10^−2^	3.11 × 10^−5^	6.14 × 10^−7^	4.64 × 10^−6^	9.33 × 10^−6^	9.10 × 10^−5^	6.75 × 10^−4^	5.01 × 10^−4^	4.51 × 10^−4^
PLY11	3.07 × 10^−1^	7.94 × 10^−2^	3.82 × 10^−5^	8.90 × 10^−7^	6.27 × 10^−6^	8.50 × 10^−6^	5.99 × 10^−5^	1.96 × 10^−4^	5.17 × 10^−4^	5.43 × 10^−4^
PLY12	2.34 × 10^−1^	2.67 × 10^−2^	4.04 × 10^−5^	5.02 × 10^−7^	4.72 × 10^−6^	8.28 × 10^−6^	5.51 × 10^−5^	2.95 × 10^−4^	3.36 × 10^−4^	3.34 × 10^−4^
PLY13	3.42 × 10^−1^	8.86 × 10^−2^	4.14 × 10^−5^	1.09 × 10^−7^	5.93 × 10^−6^	1.39 × 10^−5^	2.32 × 10^−4^	7.44 × 10^−4^	5.29 × 10^−4^	5.28 × 10^−4^
Mean	2.81 × 10^−1^	8.11 × 10^−2^	3.50 × 10^−5^	7.71 × 10^−7^	5.45 × 10^−6^	8.95 × 10^−6^	1.17 × 10^−4^	5.38 × 10^−4^	5.84 × 10^−4^	4.96 × 10^−4^

Boldface: The EDI value in this study was above the RfD value; PNY1–PNY13: The young tea samples corresponding to soil samples (PT1–PT13); PLY1–PLY13: The mature tea samples corresponding to soil samples (PT1–PT13).

**Table 6:** Target hazard quotient (THQ) and hazard index (HI) values of metals for adults due to the consumption of tea leaves in Puan tea growing, Guizhou Province.

Sample ID	THQ										HI
	Al	Mn	Pb	Cd	Hg	As	Cr	Ni	Cu	Zn	
PNY1	9.60 × 10^−3^	5.78 × 10^−2^	2.45 × 10^−3^	5.64 × 10^−4^	2.66 × 10^−2^	1.03 × 10^−2^	2.50 × 10^−5^	1.80 × 10^−2^	3.29 × 10^−2^	5.04 × 10^−3^	0.163
PNY2	1.11 × 10^−2^	2.59 × 10^−1^	4.18 × 10^−3^	4.39 × 10^−4^	2.62 × 10^−2^	4.53 × 10^−2^	6.70 × 10^−5^	2.49 × 10^−2^	2.93 × 10^−2^	5.22 × 10^−3^	0.406
PNY3	1.07 × 10^−2^	3.37 × 10^−1^	2.16 × 10^−3^	3.89 × 10^−4^	1.37 × 10^−2^	8.14 × 10^−3^	2.07 × 10^−5^	2.11 × 10^−2^	2.58 × 10^−2^	4.89 × 10^−3^	0.424
PNY4	1.27 × 10^−2^	8.78 × 10^−2^	1.59 × 10^−3^	3.51 × 10^−4^	1.76 × 10^−2^	1.01 × 10^−2^	2.55 × 10^−5^	2.30 × 10^−2^	2.38 × 10^−2^	4.83 × 10^−3^	0.182
PNY5	2.53 × 10^−2^	1.60 × 10^−1^	1.78 × 10^−3^	1.15 × 10^−3^	1.50 × 10^−2^	1.47 × 10^−2^	2.61 × 10^−5^	2.26 × 10^−2^	3.56 × 10^−2^	6.15 × 10^−3^	0.283
PNY6	1.04 × 10^−2^	2.47 × 10^−1^	1.48 × 10^−3^	2.63 × 10^−4^	1.37 × 10^−2^	7.55 × 10^−3^	3.30 × 10^−5^	3.35 × 10^−2^	2.19 × 10^−2^	5.30 × 10^−3^	0.341
PNY7	1.54 × 10^−2^	6.69 × 10^−2^	1.78 × 10^−3^	5.64 × 10^−4^	6.01 × 10^−3^	8.44 × 10^−3^	2.13 × 10^−5^	2.02 × 10^−2^	2.11 × 10^−2^	5.87 × 10^−3^	0.146
PNY8	1.54 × 10^−2^	2.54 × 10^−1^	1.12 × 10^−3^	4.89 × 10^−4^	6.01 × 10^−2^	7.65 × 10^−3^	1.81 × 10^−5^	2.16 × 10^−2^	2.38 × 10^−2^	4.53 × 10^−3^	0.335
PNY9	1.73 × 10^−2^	1.19 × 10^−1^	2.17 × 10^−3^	7.27 × 10^−4^	2.02 × 10^−2^	1.01 × 10^−2^	6.38 × 10^−5^	4.25 × 10^−2^	2.48 × 10^−2^	4.61 × 10^−3^	0.242
PNY10	1.50 × 10^−2^	1.48 × 10^−1^	1.70 × 10^−3^	4.64 × 10^−4^	1.72 × 10^−2^	2.10 × 10^−2^	2.93 × 10^−5^	2.99 × 10^−2^	2.03 × 10^−2^	4.83 × 10^−3^	0.259
PNY11	1.34 × 10^−2^	2.42 × 10^−1^	2.01 × 10^−3^	3.76 × 10^−4^	1.50 × 10^−2^	1.52 × 10^−2^	2.71 × 10^−5^	3.36 × 10^−2^	2.09 × 10^−2^	5.62 × 10^−3^	0.348
PNY12	1.23 × 10^−3^	7.73 × 10^−2^	2.02 × 10^−3^	1.50 × 10^−4^	2.79 × 10^−2^	1.79 × 10^−2^	5.75 × 10^−5^	2.91 × 10^−2^	2.62 × 10^−2^	5.78 × 10^−3^	0.199
PNY13	1.04 × 10^−2^	9.97 × 10^−2^	1.35 × 10^−3^	3.89 × 10^−4^	3.65 × 10^−2^	7.25 × 10^−3^	1.76 × 10^−5^	2.11 × 10^−2^	2.27 × 10^−2^	4.38 × 10^−3^	0.204
Mean	1.38 × 10^−2^	1.66 × 10^−1^	1.98 × 10^−3^	4.86 × 10^−4^	1.86 × 10^−2^	1.41 × 10^−2^	3.32 × 10^−5^	2.62 × 10^−2^	2.53 × 10^−2^	5.16 × 10^−3^	0.272
PLY1	1.65 × 10^−1^	1.6 × 10^−1^	9.18 × 10^−3^	7.77 × 10^−4^	1.93 × 10^−2^	2.32 × 10^−2^	1.17 × 10^−4^	2.77 × 10^−2^	2.22 × 10^−2^	1.74 × 10^−3^	0.429
PLY2	2.23 × 10^−1^	9.08 × 10^−1^	8.88 × 10^−3^	9.03 × 10^−4^	2.23 × 10^−2^	2.91 × 10^−2^	9.52 × 10^−5^	4.05 × 10^−2^	1.76 × 10^−2^	1.55 × 10^−3^	**1.251**
PLY3	2.61 × 10^−1^	**13.76 × 10^−1^**	8.82 × 10^−3^	8.40 × 10^−4^	2.02 × 10^−2^	3.57 × 10^−2^	3.94 × 10^−5^	1.78 × 10^−2^	1.38 × 10^−2^	1.50 × 10^−3^	**1.736**
PLY4	3.26 × 10^−1^	3.58 × 10^−1^	1.32 × 10^−3^	7.02 × 10^−4^	3.01 × 10^−2^	2.82 × 10^−2^	6.06 × 10^−5^	3.19 × 10^−2^	1.96 × 10^−2^	1.72 × 10^−3^	0.810
PLY5	2.69 × 10^−1^	4.36 × 10^−1^	1.24 × 10^−2^	7.52 × 10^−4^	2.92 × 10^−2^	3.30 × 10^−2^	1.06 × 10^−4^	2.60 × 10^−2^	2.21 × 10^−2^	1.89 × 10^−3^	0.830
PLY6	2.26 × 10^−1^	8.27 × 10^−1^	1.01 × 10^−2^	5.89 × 10^−4^	3.18 × 10^−2^	2.66 × 10^−2^	6.12 × 10^−5^	1.57 × 10^−2^	9.24 × 10^−3^	1.33 × 10^−3^	**1.149**
PLY7	3.68 × 10^−1^	2.32 × 10^−1^	8.87 × 10^−3^	7.40 × 10^−4^	3.82 × 10^−2^	3.05 × 10^−2^	6.28 × 10^−5^	3.79 × 10^−2^	1.69 × 10^−2^	2.44 × 10^−3^	0.736
PLY8	2.26 × 10^−1^	6.90 × 10^−1^	5.85 × 10^−3^	8.90 × 10^−4^	1.85 × 10^−2^	1.88 × 10^−2^	5.64 × 10^−5^	2.17 × 10^−2^	1.06 × 10^−2^	1.58 × 10^−3^	0.994
PLY9	3.99 × 10^−1^	6.24 × 10^−1^	7.26 × 10^−3^	7.27 × 10^−4^	3.69 × 10^−2^	2.12 × 10^−2^	1.24 × 10^−4^	3.49 × 10^−2^	1.07 × 10^−2^	1.56 × 10^−3^	**1.137**
PLY10	3.07 × 10^−1^	5.28 × 10^−1^	8.64 × 10^−3^	6.14 × 10^−4^	2.32 × 10^−2^	3.01 × 10^−2^	6.06 × 10^−5^	3.38 × 10^−2^	1.25 × 10^−2^	1.50 × 10^−3^	0.946
PLY11	3.07 × 10^−1^	5.67 × 10^−1^	1.06 × 10^−2^	8.90 × 10^−4^	3.13 × 10^−2^	2.74 × 10^−2^	3.99 × 10^−5^	9.78 × 10^−3^	1.29 × 10^−2^	1.81 × 10^−3^	0.969
PLY12	2.34 × 10^−1^	1.90 × 10^−1^	1.12 × 10^−2^	5.02 × 10^−4^	2.36 × 10^−2^	2.67 × 10^−2^	3.67 × 10^−5^	1.48 × 10^−2^	8.41 × 10^−3^	1.11 × 10^−3^	0.511
PLY13	3.42 × 10^−1^	6.33 × 10^−1^	1.15 × 10^−2^	1.09 × 10^−3^	2.96 × 10^−2^	4.50 × 10^−2^	1.55 × 10^−4^	3.72 × 10^−2^	1.32 × 10^−2^	1.76 × 10^−3^	**1.114**
Mean	2.81 × 10^−1^	5.97 × 10^−1^	9.73 × 10^−3^	7.71 × 10^−4^	2.73 × 10^−2^	2.89 × 10^−2^	7.80 × 10^−5^	2.69 × 10^−2^	1.46 × 10^−2^	1.65 × 10^−3^	0.970

Boldface: The THQ and HI values were above one; PNY1–PNY13: the young tea leaves samples corresponding to soil samples (PT1–PT13); PLY1–PLY13: the mature tea samples corresponding to soil samples (PT1–PT13).
